# Nonsense variant in *COL7A1* causes recessive dystrophic epidermolysis bullosa in Central Asian Shepherd dogs

**DOI:** 10.1371/journal.pone.0177527

**Published:** 2017-05-11

**Authors:** Julia Niskanen, Kati Dillard, Meharji Arumilli, Elina Salmela, Marjukka Anttila, Hannes Lohi, Marjo K. Hytönen

**Affiliations:** 1 Department of Veterinary Biosciences, University of Helsinki, Helsinki, Finland; 2 Research Programs Unit, Molecular Neurology, University of Helsinki, Helsinki, Finland; 3 The Folkhälsan Institute of Genetics, Helsinki, Finland; 4 Pathology Unit, Finnish Food Safety Authority, Evira, Helsinki, Finland; 5 Department of Biosciences, University of Helsinki, Helsinki, Finland; Ecole normale superieure de Lyon, FRANCE

## Abstract

A rare hereditary mechanobullous disorder called epidermolysis bullosa (EB) causes blistering in the skin and the mucosal membranes. To date, nineteen EB-related genes have been discovered in human and other species. We describe here a novel EB variant in dogs. Two newborn littermates of Central Asian Shepherd dogs with severe signs of skin blistering were brought to a veterinary clinic and euthanized due to poor prognosis. In post-mortem examination, the puppies were shown to have findings in the skin and the mucosal membranes characteristic of EB. A whole-genome sequencing of one of the affected puppies was performed to identify the genetic cause. The resequencing data were filtered under a recessive model against variants from 31 other dog genomes, revealing a homozygous case-specific nonsense variant in one of the known EB-causing genes, *COL7A1* (c.4579C>T, p.R1527*). The variant results in a premature stop codon and likely absence of the functional protein in the basement membrane of the skin in the affected dogs. This was confirmed by immunohistochemistry using a COL7A1 antibody. Additional screening of the variant indicated full penetrance and breed specificity at ~28% carrier frequency. In summary, this study reveals a novel *COL7A1* variant causing recessive dystrophic EB and provides a genetic test for the eradication of the disease from the breed.

## Introduction

Epidermolysis bullosa (EB) belongs to a group of rare inherited skin conditions characterized by development of blisters in the dermo-epidermal junction. EB is divided into several subtypes based on structural changes in the skin, the mode of inheritance, and clinical, microscopic and immunohistochemical findings (1). The four main types include EB simplex (EBS), junctional EB (JEB), dystrophic EB (DEB) and Kindler syndrome (KS), which differ in their phenotype and genotype; a common feature of all of them is the fragility of the skin and mucosae due to defects in the structural proteins of the basement membrane zone [1]. The level of cleavage between the dermis and the epidermis varies by the subtype, and is located in the basal or suprabasal cell layer in EBS, in the lamina lucida in JEB or below the lamina densa in DEB [2]. Multiple levels of cleavage within basal keratinocytes, lamina lucida or sublamina densa region are found in the KS [2]. EB is clinically highly heterogeneous with subtype-specific symptoms but distinguishing features include blisters and skin erosions, milia, nail defects, granulation tissue, keratoderma and dyspigmentation [1]. The disease onset varies between birth and early infancy [1].

A wide range of genetic causes for EB have been identified, reflecting the heterogeneous clinical picture. The list of nineteen known recessive or dominant EB genes include *COL7A1*, *COL17A1*, *DSP*, *DST*, *EXPH5*, *FERMT1*, *ITGA3*, *ITGA6*, *ITGB4*, *JUP*, *KLHL24*, *KRT5*, *KRT14*, *LAMA3*, *LAMB3*, *LAMC2*, *PLEC*, *PKP1* and *TGM5* [3,4]. To date, defects in four of them have been linked to different types of canine EB: a premature stop codon in *PKP1* causing ectodermal dysplasia-skin fragility syndrome [5], a nonsense mutation in *PLEC* causing EBS [6], an insertion in *LAMA3* causing JEB [7] and a missense mutation in *COL7A1* causing a mild form of DEB [8]. Additional EB cases with unknown molecular background have been described in German Shorthaired Pointers and mixed-breed dogs with JEB as well as in Akita Inu with DEB [9]. We describe here a novel recessive and severe form of dystrophic EB in Central Asian Shepherd (CAS) dogs with a nonsense variant in *COL7A1*.

## Results

### Histological analysis reveals typical features of EB

Two CAS dogs from a litter of eight puppies were brought to a veterinary clinic soon after birth due to severe skin lesions, blisters and ulcers on feet, ears, muzzle and oral mucosa. Epidermolysis bullosa was suspected and the affected dogs were euthanized due to a poor prognosis and sent for necropsy.

**Fig 1:**
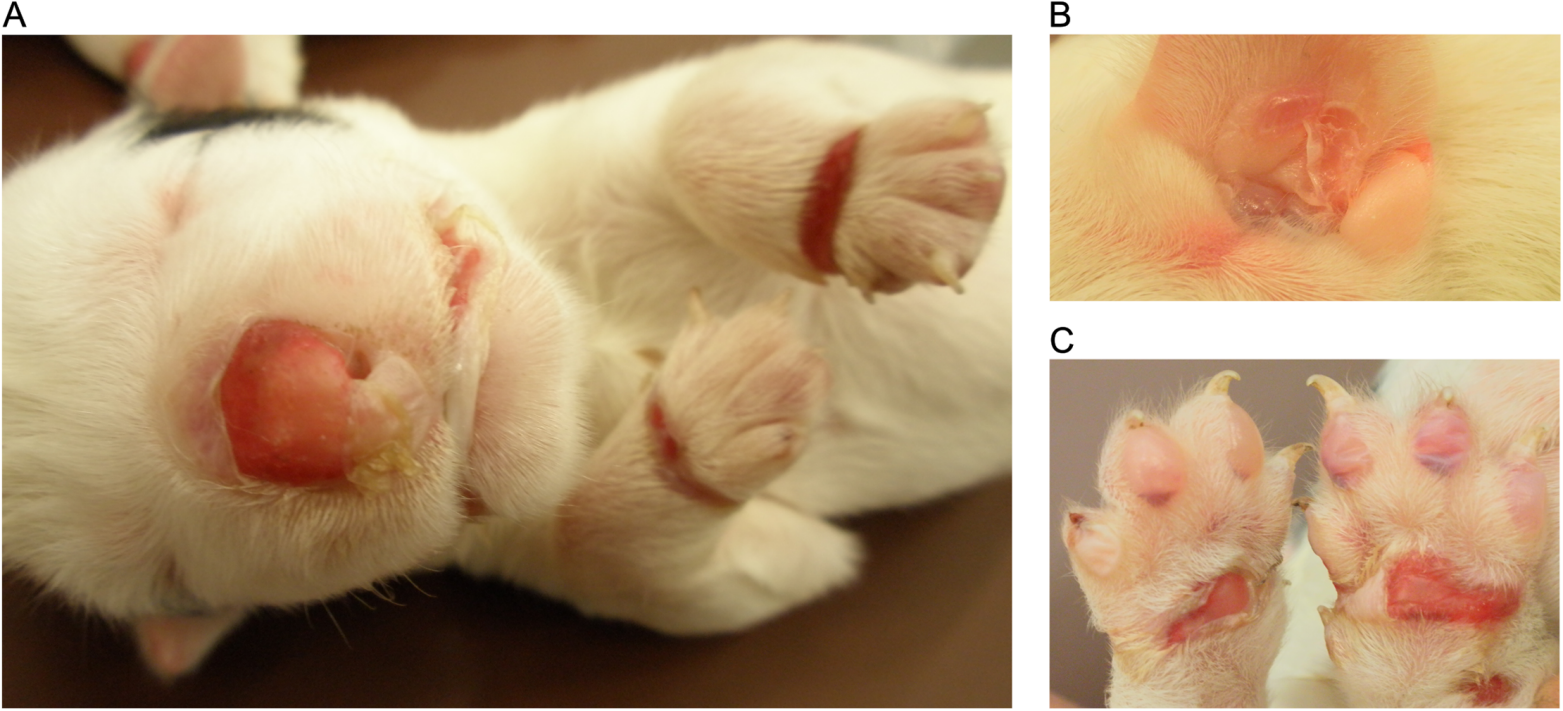
The macroscopic changes of epidermolysis bullosa in the affected puppy. (A) Large erupted blisters were present on the nasal plane, lips and dorsal skin of the front paws. (B) Large intact and erupted blisters were also present in the inner ear lobe. (C) Numerous intact bullae were found on the digital pads and the erupted blisters of metacarpal and carpal pads resulted in the formation of large ulcers.

**Fig 2:**
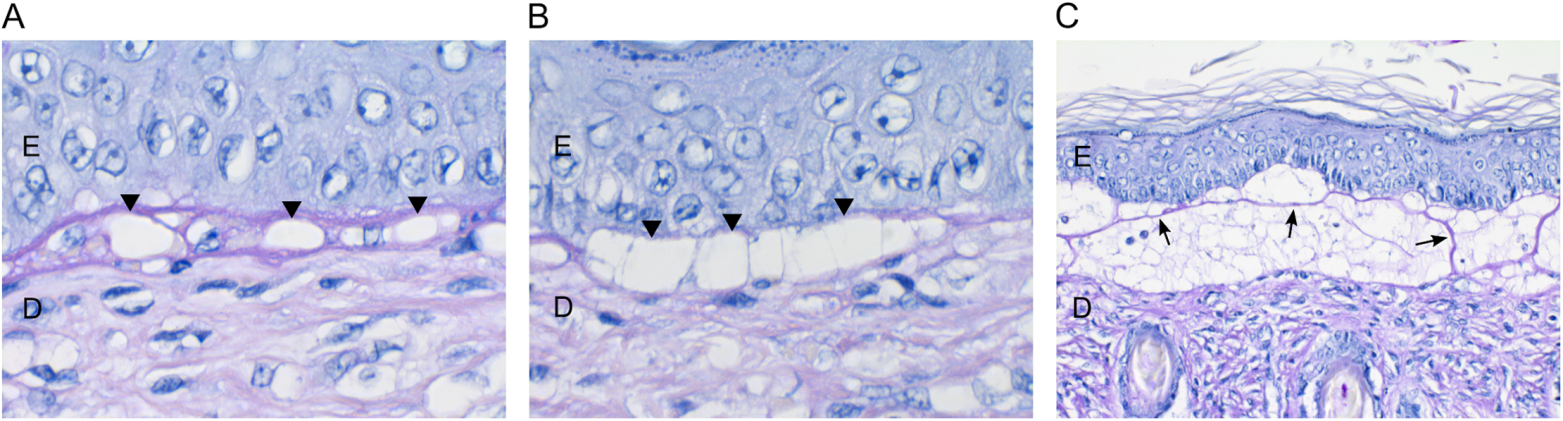
Histological stainings of the haired skin of the inner ear lobe by periodic acid–Schiff (PAS). (A) Small vacuoles at the basement membrane (arrowheads) (63X). (B) Larger, confluent vacuoles result in a split along the basement membrane zone (arrowheads) (63X). (C) Large subepidermal cleft with PAS-positive material, suggestive of basement membrane, on the roof and floor of the cleft and strands crossing the bulla (arrows) (20X). Epidermis is denoted with “E” and dermis with “D”.

Both puppies had multiple vesicles, bullae and ulcers in the oral and oropharyngeal mucosa, inner pinnae, dorsal plane of the nose, ventral abdomen, external genitalia, metacarpal and metatarsal skin and footpads (Fig 1). Histologically, there was separation at the dermal-epidermal junction with cleft formation. Small and large vacuoles were detected at the basement membrane (Fig 2A and 2B). Some of the large vacuoles were confluent, resulting in a split at the basement membrane. PAS-positive material, suggestive of basement membrane, was found on the roof and/or floor in the bullae and strands crossing the bullae were seen in the larger clefts (Fig 2C). The larger clefts included numerous mononuclear cells, neutrophils, necrotic debris and erythrocytes denoting secondary inflammation. No specific macroscopic or histological changes were present in the brain, the respiratory and gastrointestinal tract, heart, liver, spleen, pancreas, kidneys, thyroid and adrenal glands and gonads.

### COL7A1 truncation confirms dystrophic EB

**Fig 3:**
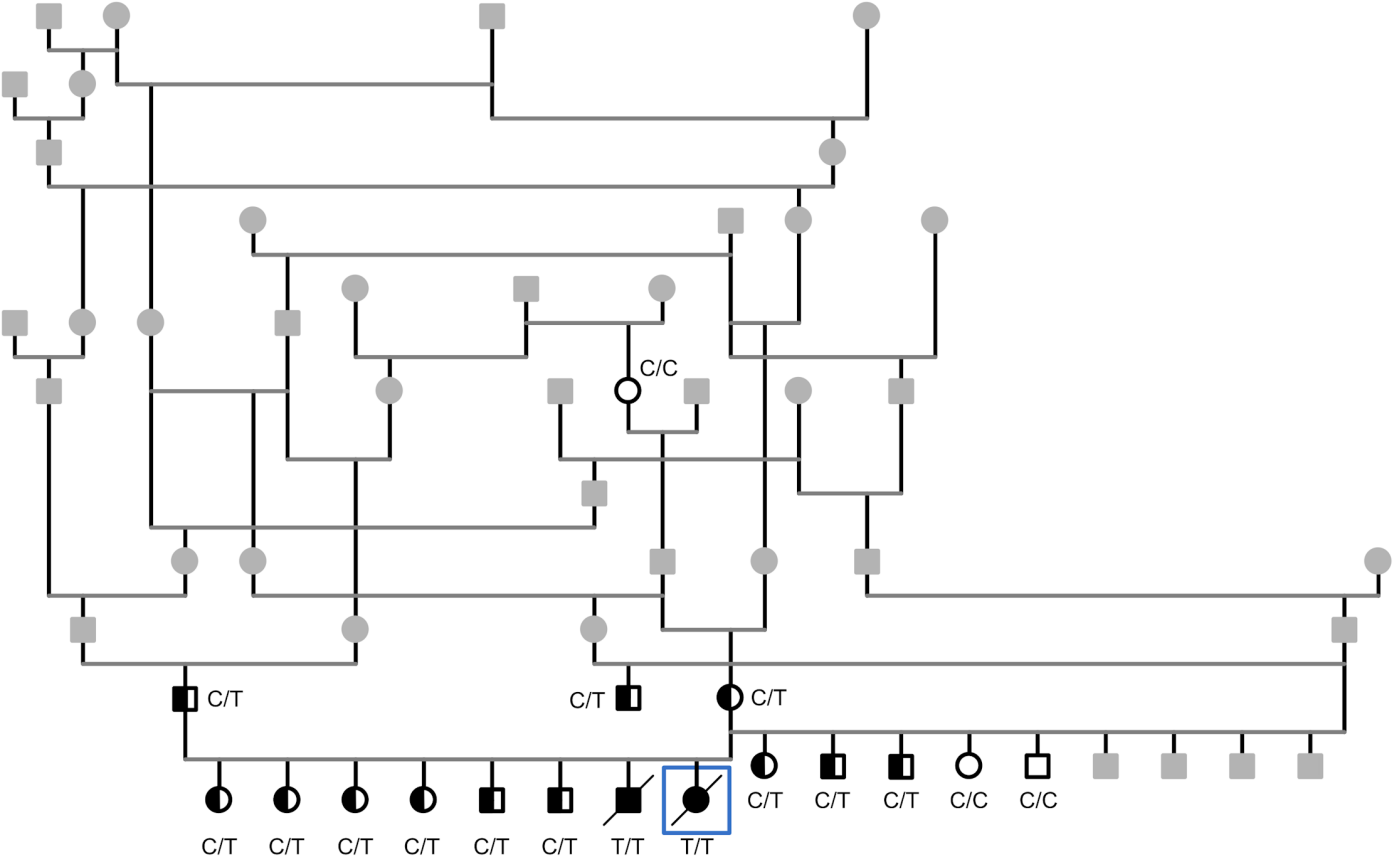
A pedigree of the affected puppies with a suspected recessive mode of inheritance. The *COL7A1* variant genotype is denoted for individuals that were tested. White symbol denotes wild type (C/C), half-filled heterozygous carrier (C/T) and black symbol homozygous mutant (T/T), while grey denotes that no sample was available for testing. The whole genome was sequenced from the affected dog surrounded with a square.

**Fig 4:**
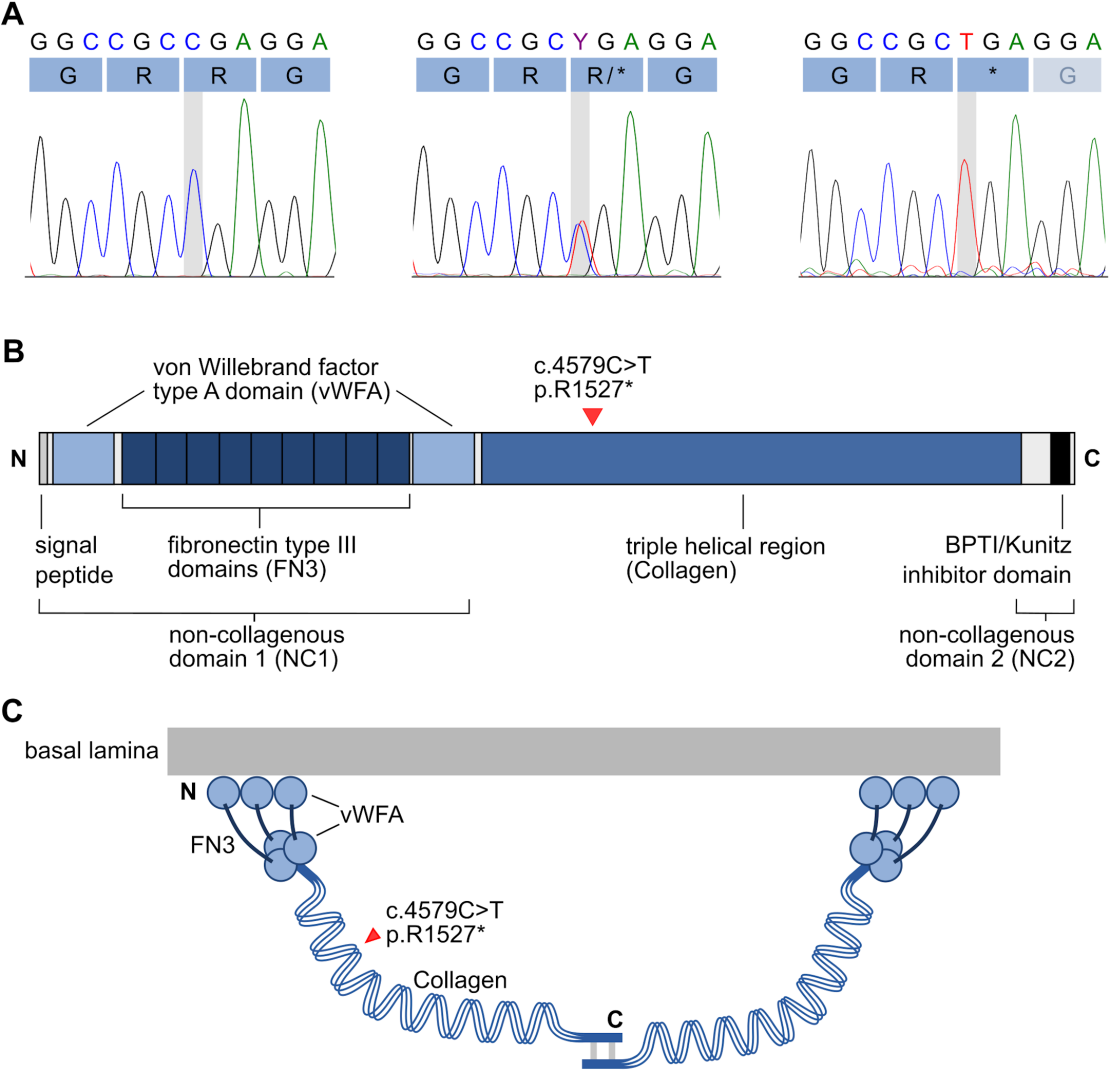
Chromatograms of the *COL7A1* c.4579C>T variant and a schematic representation of the collagen VII protein. (A) Three genotypes are present in the Central Asian Shepherd Dog cohort: wild type C/C, heterozygous carrier C/T and homozygous mutant T/T. The mutation causes a change from arginine (CGA) to a stop codon (TGA). (B) The domain structure of collagen VII. The substitution site p.R1527* is located near the beginning of the characteristic triple-helical domain. (C) A schematic presentation of the dimerization of collagen VII homotrimers. If the abnormal COL7A1 is translated the p.R1527* substitution results in severe truncation of the protein, preventing the dimerization of collagen VII at the C-terminal end.

EB is inherited either in a recessive or dominant manner. A recessive mode of inheritance was suspected in the CAS pedigree as the two severely affected CAS dogs were born to healthy parents (Fig 3). The whole genome of one affected puppy was sequenced with an average coverage of 13X. Altogether 6 880 938 variants were called, of which 2 776 951 were homozygous. The variants were filtered against whole-genome data from 31 other dogs (Table 1) under recessive model. This decreased the number of homozygous coding variants to 401 and only one of them was found in the known EB genes, a nonsense variant at chr20:40,532,043 in the *COL7A1* gene (c.4579C>T, p.R1527*) (Fig 4). This variant results in a premature stop codon in the 45th exon (p.R1527*) and a predicted early truncation of COL7A1 (Fig 4).

**Table 1:** Summary of the unaffected control dogs used in the whole-genome variant filtering study.

Breed	Number of dogs
Border Collie	22
Alaskan Malamute	1
Dalmatian	1
Karelian Bear Dog	1
Leonberger	1
White Shepherd Dog	1
Dandie Dinmont Terrier	1
Swedish Vallhund	1
Welsh Springer Spaniel	1

The identified *COL7A1* variant was selected as a prime candidate for EB in the CAS dogs for three reasons. First, COL7A1 defects are known to cause recessive dystrophic EB in human and animals [9,10]. Second, the identified homozygous nonsense variant is predicted to result in the severe truncation of the COL7A1 protein, which very likely renders it non-functional. Finally, none of the other genes with coding variants are known to cause EB in any species and appeared therefore unlikely to be causal. To understand the penetrance, prevalence and specificity of the mutation, we genotyped the identified *COL7A1* variant in the second affected dog, the unaffected littermates, the parents, and additional 37 non-EB CAS dogs available in our biobank. The second affected dog was also homozygous for the nonsense variant while the parents and the unaffected littermates were carriers, indicating a complete segregation of the mutation with the disease. The mutation was absent in 143 dogs from related breeds (Table 2), and found only in the CAS breed at 27.7% carrier frequency.

**Table 2:** Summary of study cohorts used in the COL7A1 c.4579C>T variant screening.

Breed	Number of dogs	Number of carriers
Central Asian Shepherd Dog	47	13
Caucasian Shepherd Dog	39	0
South Russian Ovcharka	3	0
Kuvasz	6	0
Slovakian Chuvach	19	0
Tibetan Mastiff	76	0

**Fig 5:**
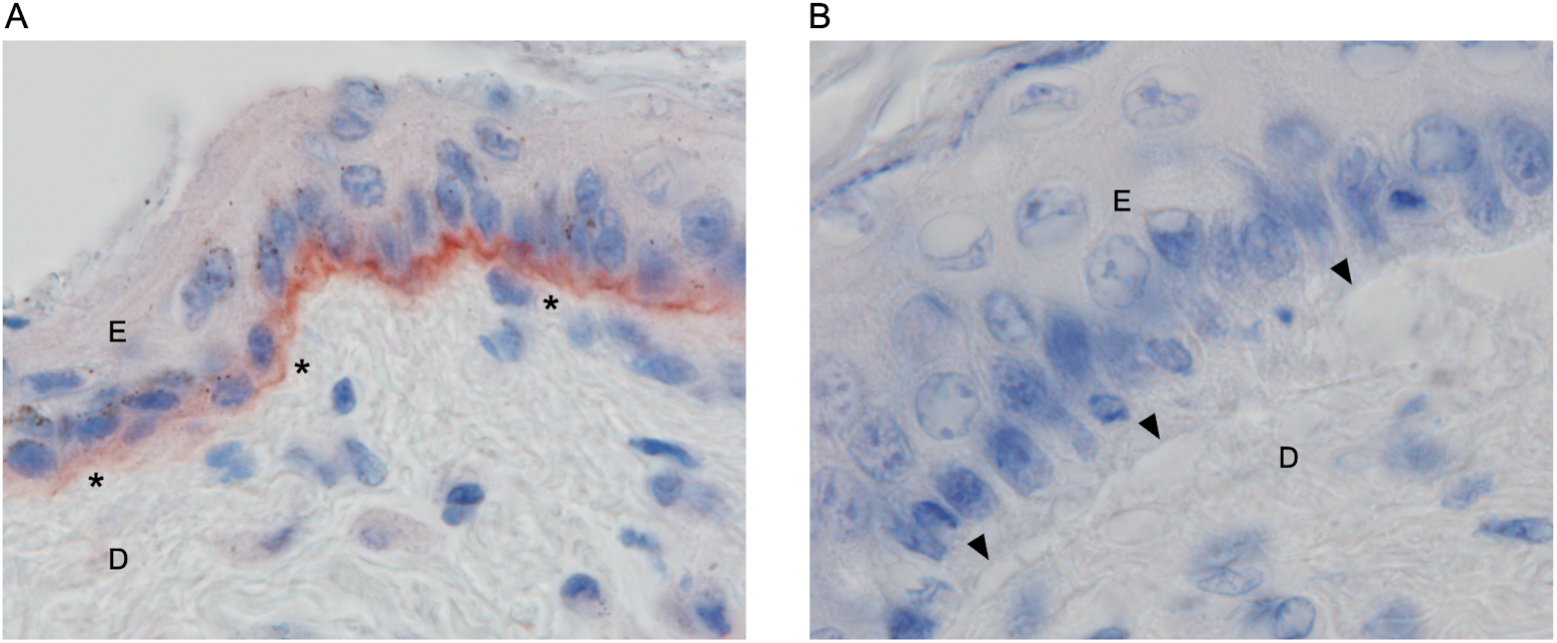
Immunohistochemical staining for collagen VII. (A) The basement membrane zone between the epidermis and dermis is positive for collagen VII in the haired skin of the flank of an unaffected control. (100X) (B) The basement membrane zone between the intact epidermis and dermis and the roof and floor of the vacuoles during early cleft formation are negative for collagen VII in the haired skin of the inner ear lobe of an affected puppy (100X). Epidermis is denoted with “E” and dermis with “D”.

To study the effect of the truncation mutation on the expression of COL7A1, we performed immunohistochemical staining in the skin samples of the affected puppies and age-matched controls using a COL7A1 antibody. In the control skin, a strong linear positive collagen VII staining was detected in the basement membrane zone of the epidermis (Fig 5A). In the affected puppies, the basement membrane zone was completely negative for collagen VII (Fig 5B). These results demonstrate the absence of functional COL7A1 in the affected dogs.

## Discussion

We discovered a novel pathological *COL7A1* variant that results in a premature stop codon and subsequently causes recessive dystrophic epidermolysis bullosa (RDEB) in the CAS breed. RDEB is a subtype of EB that is characterized by generalized blistering and scarring of the skin as well as extracutaneous complications, such as failure to thrive, multifactorial anemia, pseudosyndactyly and esophageal strictures [1,11]. The clinical symptoms of the two affected CAS dogs were very similar to those of human RDEB, especially in terms of the severity, the onset at birth, failure to thrive, and the extent of the skin lesions. The macroscopic and histological changes in the skin were also typical of EB. In human dystrophic EB patients, over 300 *COL7A1* mutations have been found, and they include nonsense, missense, deletion, insertion, insertion-deletion, splice-site and regulatory mutations [10]. Generally, the type and position of the mutation correlates with the severity of the phenotype, and premature stop codons in the triple-helical domain often have serious effects [10].

In the case of the CAS dogs, the premature stop codon in *COL7A1* results in the absence of functional COL7A1. It is possible that the abnormal transcript either undergoes nonsense-mediated decay or, if stable enough to be translated, produces a severely truncated COL7A1 protein, which in turn is unstable, or fails to be deposited at the dermal-epidermal junction. The lack of COL7A1 expression was confirmed by IHC staining in the skin of the affected dogs. During maturation, three COL7A1 subunits are assembled into collagen VII homotrimers, the NC2 domain in the C-terminus is proteolytically processed, and two homotrimers are linked from their C-terminal ends with disulfide bonds to form dimers [12]. The possible truncated COL7A1 would lack C-terminal ends, which are required to link the dimers, and therefore the assembly of the anchoring fibrils would be compromised in the affected dogs.

The CAS disease represents a severe form of RDEB, consistent with the nonsense *COL7A1* variant. Another canine RDEB model has been previously described in Golden Retrievers with a missense mutation in *COL7A1* (c.5761G>A, p.G1906S) [8,13]. The affected dogs were reported to suffer from a mild form of the disorder, however, a more detailed description was not provided for comparison with the CAS dogs.

No cure for EB exists. Multiple treatments have been studied, including gene, recombinant protein and cell therapies, gene editing and induced pluripotent stem cells (iPSC) [14–21]. Regarding RDEB specifically, the goal of gene therapy would be to restore the collagen VII function in epithelial tissues. The Golden Retriever RDEB model has been utilized to study cell therapy, such as the clinical effects of *ex vivo* corrected keratinocyte grafts [8,22]. The severity of EB in the CAS dogs challenges their utilization in intervention studies, and therefore, the major impact of our gene discovery is that it enables an efficient identification of the carrier dogs and subsequent eradication of this serious disease through revised breeding programs.

## Materials and methods

### Ethics statement

Sample collection in Finland was ethically approved by the Animal Ethics Committee of State Provincial Office of Southern Finland (ESAVI/7482/04.10.07/2015). All samples were collected from privately owned dogs by owner’s consent.

### Study cohort

A CAS litter of eight puppies was born at term. Two puppies (male and female) were euthanized at owner’s request soon after birth due to severe lesions in the haired skin and oral mucosa, and the affected puppies were taken to necropsy. The puppies were euthanized by a veterinarian, and the euthanization consisted of intramuscular sedation followed by administration of a lethal overdose of intravenous anesthetic. During necropsy, a liver sample was collected and frozen for later DNA isolation. EDTA blood was collected from the healthy littermates, parents and other unaffected dogs from the breed. The samples were stored at -20°C. Genomic DNA was extracted using the semi-automated Chemagen extraction robot (PerkinElmer ChemagenTechnologie GmbH). DNA concentration was determined either with NanoDrop ND-1000 UV/Vis Spectrophotometer or Qubit 3.0 Fluorometer (Thermo Fisher Scientific Inc). Pedigree data were obtained from the public databases of the Finnish Kennel Club (http://jalostus.kennelliitto.fi/) and the Estonian Kennel Club (http://register.kennelliit.ee/). The pedigree was constructed with the AncesTrim software [23] and rendered with GenoPro (http://www.genopro.com/).

### Necropsy

A full necropsy was performed on both puppies. Tissue samples of the skin, oral mucosa, internal organs and brain were collected for histological examination. The tissue samples were formalin fixed, routinely processed, paraffin embedded, sectioned at 4 μm thickness and stained with hematoxylin and eosin (HE). Skin samples were also stained with the periodic acid–Schiff (PAS) stain to delineate the zone of separation at the dermo-epidermal junction. Healthy control skin samples for IHC staining were collected at necropsy from two puppies of different breeds that had died due to other diseases.

### Whole-genome sequencing

We performed whole-genome sequencing on one affected Central Asian Shepherd dog. A fragment library with a median insert size of 400 bp was prepared and sequenced on Illumina HiSeq2500 to generate paired-end reads of 2*125 bp. Mapping, variant calling and subsequent data analysis was performed as previously described in Hytönen et al. 2016 [24].

### Genomic DNA analysis

Cohorts of 47 Central Asian Shepherd dogs and several related breeds, including 39 Caucasian Shepherd Dogs, 76 Tibetan Mastiffs, 3 South Russian Ovcharkas, 6 Kuvasz and 19 Slovakian Chuvach, were genotyped for the *COL7A1* c.4579C>T variant. Genotyping of the variant was performed by standard PCR with the following primers: 5’-CTCTGCTCTTCTTCCCCAGG-3’ and 5’-CCTCCCTCTGCTTACCACTG-3’. The amplified PCR products were sequenced with a capillary sequencer and the sequence data were analyzed using Sequencher 5.1 (GeneCodes).

### Immunohistochemistry

The skin samples of two affected puppies were sectioned at 4 μm thickness and deparaffinized. Antigen retrieval was done with Proteinase K for 5 minutes at 37°C. The primary collagen VII antibody (Monoclonal Anti-Collagen, Type VII Antibody, clone LH7.2, SAB4200686, Sigma-Aldrich, USA) was incubated for two hours at 37°C with a dilution of 1:50. The tissue sections were stained with UltraVision LP Detection System HRP polymer and AEC chromogen kit (Thermo Fischer Scientific Inc., USA). The staining was performed according to the protocol of the kit without the hydrogen peroxide block step. Skin samples collected at necropsy from two 0 to 2-day-old puppies with normal skin were used as controls.
